# Sonographic signs of deep infiltrative endometriosis among women submitted to routine transvaginal sonography: clinical and imaging aspects

**DOI:** 10.31744/einstein_journal/2022AO0086

**Published:** 2022-11-09

**Authors:** Luciana Cristina Pasquini Raiza, Paulo Homem de Mello Bianchi, Carla de Azevedo Piccinato, Sérgio Podgaec

**Affiliations:** 1 Hospital Israelita Albert Einstein São Paulo Brazil Hospital Israelita Albert Einstein, São Paulo, SP, Brazil.; 2 Universidade de São Paulo Hospital das Clínicas Faculdade de Medicina São Paulo Brazil Hospital das Clínicas, Faculdade de Medicina, Universidade de São Paulo, São Paulo, SP, Brazil.

**Keywords:** Endometriosis, Endometrial neoplasms, Prevalence, Ultrasonography

## Abstract

**Objective:**

To evaluate the prevalence of sonographic signs suggestive of deep infiltrative endometriosis and endometriomas in patients referred for transvaginal sonography as part of a routine annual gynecological evaluation. We also describe the clinical and imaging aspects associated with the incidental findings of endometriosis.

**Methods:**

This was a retrospective observational study including women (n=339; aged 18-56 years) referred for transvaginal sonography as part of a routine gynecological evaluation (without clinical suspicion of endometriosis). Patients were asked about their symptoms. In addition, they were systematically checked by an experienced radiologist for sonographic signs of deep infiltrative endometriosis (hypoechoic nodules or tissue thickening, with regular or irregular margins) in the retrocervical area, vaginal fornix, rectosigmoid junction, and bladder, as well as for ovarian endometriomas (cysts with thick walls and hypoechogenic content).

**Results:**

Signs suggestive of deep infiltrative endometriosis or endometriomas were identified in 27 of the 339 women (8.0%; 95%CI: 5.1-10.8). Endometriomas were observed in 8 patients (2.4%; 95%CI: 0.7-4.0); 23 women had signs of lesions in the retrocervical area (6.8%; 95%CI: 4.1-9.5), 3 in the rectum and sigmoid colon (0.9%; 95%CI: 0-1.9), and 1 in the vagina (0.3%; 95%CI: 0-0.9). Six patients (1.8%) had signs of endometriosis at more than one site, and thirteen were asymptomatic. There were no significant differences in symptomatology between women with and without sonographic signs of deep infiltrative endometriosis.

**Conclusion:**

Routine transvaginal sonography offers an opportunity to search for signs of deep infiltrative endometriosis in oligosymptomatic women particularly those not previously suspected to have endometriosis.

## INTRODUCTION

Although endometriosis is associated with painful symptoms, infertility, and cyclic bowel and urinary symptoms,^(1,2)^ which could have a significant impact on the woman’s quality of life, the mean time from symptom onset to definitive diagnosis is usually several years.^(3)^ Therefore, difficulties associated with endometriosis diagnosis must be addressed urgently. The fact that the symptoms are not very specific and that there is not much clinical suspicion are important features. However, they probably only explain part of the delay in diagnosis.^(3)^

The gold standard diagnostic test is the direct visualization of lesions during surgery and histopathological confirmation.^(4)^ An invasive form of diagnosis, although precise, may introduce some bias and limitations to the study of the disease. For instance, the true prevalence of endometriosis in women during menacme remains unknown.^(4)^ Surgical findings in symptomatic women show that endometriosis affects up to 47% of women with infertility and up to 21% of those with chronic pelvic pain.^(5–8)^

Transvaginal sonography (TVS) has proven to be an accurate tool for detecting and staging ovarian endometriomas and deep infiltrative endometriosis (DIE). This procedure is also helpful to assist treatment decisions, surgical plan, and postoperative follow-up.^(9)^ Transvaginal sonography has been the imaging test of choice for women with gynecological symptoms for a long time. Transvaginal sonography has also been progressively used to complement gynecological physical examination during annual checkups (routine TVS or rTVS), even in asymptomatic women. However, if endometriosis has not been clinically suspected previously, it is often not actively investigated during rTVS. Moreover, most DIE lesions are probably left undetected during rTVS, except for large ovarian endometriomas.^(10)^

## OBJECTIVE

To evaluate the prevalence of sonographic signs suggestive of deep infiltrative endometriosis and endometriomas in patients referred for transvaginal sonography as part of a routine annual gynecological evaluation. We also describe the clinical and imaging aspects associated with the incidental findings of endometriosis.

## METHODS

We conducted a retrospective analysis of TVS examinations performed in women referred for pelvic examinations as part of an annual gynecological checkup over 28 months (November 2013 to February 2016) in the radiology department of a private hospital. The study was approved by the Institutional Review Board (IRB), *Hospital Israelita Albert Einstein,* CAAE: 55832216.6.0000.0071; # 1.644.294.

All examinations included in this study were performed by a radiologist with more than 5 years of experience in detecting deep DIE using TVS. However, none of the women included in the present study were referred for examination to clarify the clinical suspicion of endometriosis. Therefore, none of scans were performed after bowel preparation, which is the institution’s protocol for TVS when there is a previous suspicion of DIE.

On the day of the examination, each participant’s age, gynecological symptoms (dysmenorrhea, deep dyspareunia, chronic pelvic pain, cyclic bowel and urinary symptoms, and infertility), and history of pelvic diseases or gynecological surgery were recorded.

We excluded women who had already been diagnosed with endometriosis or those who had been previously investigated for it, as well as postmenopausal women.

### Examination protocol

Examinations were performed using a Toshiba Aplio™ XG or Toshiba Aplio™ 500 machine with an endocavitary probe (8.8-3.6MHz). Scans were performed in all phases of the menstrual cycle. However, in contrast to the specific protocol to detect endometriosis, no bowel preparation was performed before the examination in this study. Nonetheless, along with other gynecological findings, endometriotic lesions have been actively investigated.

Through the transvaginal route, the uterus, ovaries, vesicouterine pouch, bladder wall, retrocervical region, vaginal fornix, and rectosigmoid junction were all examined systematically.

The following sonographic signs were considered suspicious for endometriosis, as previously described: bladder - hypoechoic lesions with or without regular contours involving the muscularis or (sub) mucosa of the bladder;^(11–16)^ posterior compartment - hypoechoic solid nodules with smooth or irregular contours and hypoechoic thickening of the wall of the bowel or vagina;^(16)^ uterosacral ligaments - hypoechoic thickening with regular or irregular margins;^(16)^ vagina - hypoechoic homogeneous or inhomogeneous nodule with or without cystic areas or hypoechoic vaginal wall thickening;^(11,13,15)^ rectum, rectosigmoid junction, and sigmoid - hypoechoic thickening of the muscularis propria or hypoechoic nodules; hypoechoic thickening of the bowel.^(16–19)^

On the ovaries, the presence of a cyst with thick walls and hypoechogenic homogeneous content (“ground glass”) was considered suspicious for an endometrioma. As previously described, fluid levels, septa, and bright foci might be present.^(16,20)^

### Statistical analysis

To compare women with and without sonographic signs suggestive of endometriosis, the following statistical tests were used: χ^2^, Fisher’s exact test, Student *t*-test, and Mann-Whitney U test. Categorical variables (dysmenorrhea, deep dyspareunia, chronic pelvic pain, cyclic bowel and urinary symptoms, and infertility) were described using absolute and relative frequencies. The highest and lowest values, mean and standard deviation, median, and interquartile interval (1^st^ and 3^rd^) were used to describe the quantitative variables (age, number of lesions, and endometrioma sizes).

Data are presented with a 95% confidence interval (95%CI). All analyses were conducted using IBM SPSS Statistics for Windows, version XX (IBM Corp., Armonk, N.Y., USA), and the significance level was set at 5%.

## RESULTS

During the investigation period, 441 scans were performed on 420 women; 17 women underwent rTVS on more than one occasion during the study, and the others were evaluated only once. Eighty-one women were excluded from the study, including 55 postmenopausal women and 26 women with a previous investigation or confirmed diagnosis of endometriosis. Therefore, we analyzed the data from 339 women.

Table 1 presents the characteristics of the women included in this study. Women’s ages varied between 18 and 56 years (mean age, 35.6±8.3 years). While dysmenorrhea was reported by 45.9% of participants, infertility was reported by only 2.1% of the patients, and only 177 (52.2%) women reported having already tried to conceive.

**Table 1:** Characteristics of the women included in the study: age and symptoms

Age (years old) (±SD)	35.6 (±8.3) (range 18-56)
Dysmenorrhea, n (%)	150 (45.9)
Dyspareunia, n (%)	8 (2.4)
Chronic pelvic pain, n (%)	11 (3.4)
Cyclic bowel symptoms, n (%)	12 (3.7)
Infertility*, n (%)	7 (2.1)
No symptoms reported, n (%)	167 (54.1)
Total number of patients	339

* Only 177 women have reported already tried to conceive.

SD: standard deviation.

Signs suggestive of endometriosis were found in 27 women (8.0%; 95%CI: 5.1-10.8). The anatomical location of the lesions is described in table 2. Endometrioma sizes varied between 0.9 and 4.3cm, and the left ovary was more commonly affected (n=7) than the right one. Half of the patients in whom endometrioma was detected (n=4) also had signs of DIE.

**Table 2:** Anatomical distribution of lesions suggestive of endometriosis on routine sonographic examination

	n	95%CI
Endometrioma	8	2.4 (0.7-4.0)
Retrocervical area	23	6.8 (4.1-9.5)
Bowel	3	0.9 (0-1.9)
Vagina	1	0.3 (0-0.9)
Bladder	0	0

95%CI: 95% confidence interval.

**Figure 1:**
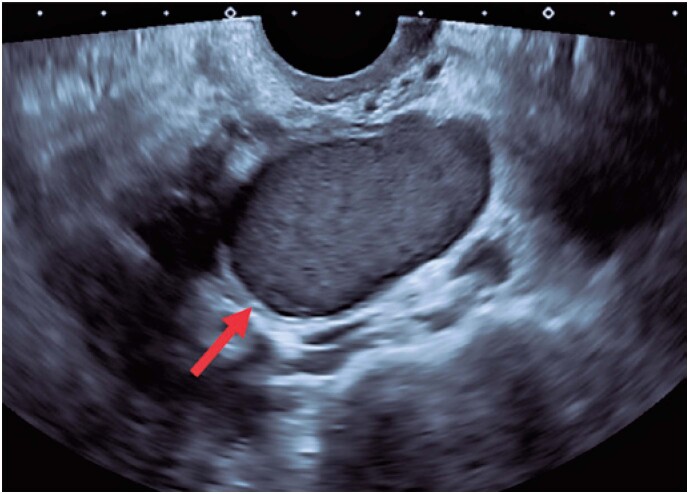
A sonographic transvaginal image of a cyst with homogeneous hypoechogenic content (“ground glass”) in the left ovary (arrow), which suggested endometrioma

**Figure 2:**
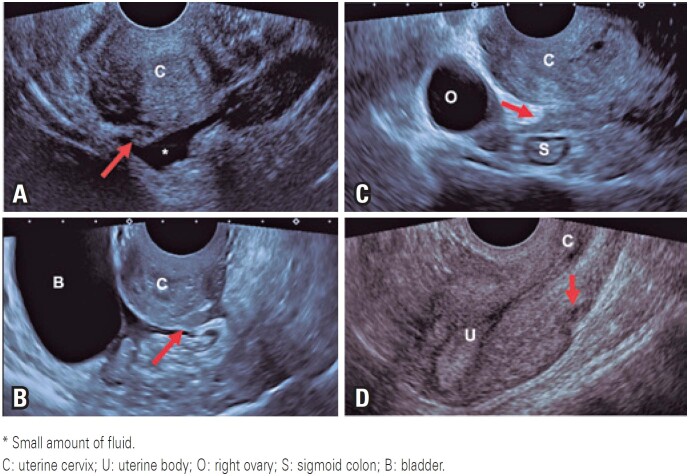
This figure shows some sonographic signs suggestive of deep infiltrative endometriosis affecting the posterior compartment (arrows) (A to C). (A) A hypoechogenic nodule at the uterine insertion of the right uterosacral ligament; (B and C) A retrocervical hypoechogenic thickening with regular (B) and irregular (C) margins; (D) Myometrium-infiltrating hypoechogenic tissue

**Figure 3:**
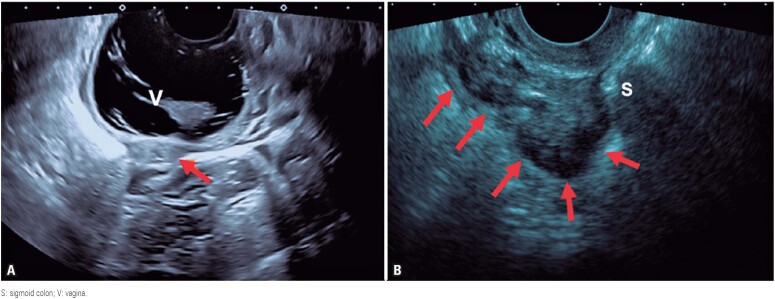
(A) A regular hypoechoic nodule (arrow) infiltrating the vaginal wall. The vagina was distended with sonographic gel for better delimitation of the lesion; (B) A transvaginal ultrasound of the sigmoid shows hypoechoic thickening of the bowel wall (arrows) infiltrating the muscular layer

Most of the patients (n=21) had only one suspected lesion (6.2%), but 6 women (1.8% of the total) had two or more suspected lesions that were detected in different anatomical sites. Only 2 women (0.6% of the sample) had lesions in 3 anatomical locations, including the ovaries, retrocervical area, and bowel. Figures 1 to 3 depict the images of some detected lesions.

Table 3 compares women with and without sonographic signs of endometriosis based on their age and whether the clinical symptoms were present.

**Table 3:** Comparison between women with and without sonographic signs suggestive of endometriosis for age and symptoms

	With TVS signs of endometriosis	Without TVS signs of endometriosis	p value
Age (±SD)	38.2 (7.2)	34.4 (8.3)	0.088
Asymptomatic, n (%)	12 (44.4)	155 (51.7)	0.472
Dysmenorrhea, n (%)	13 (48.1)	137 (45.7)	0.804
Dyspareunia, n (%)	1 (3.7)	7 (2.3)	0.502
Chronic pelvic pain, n (%)	3 (11.1)	8 (2.7)	0.053
Cyclic bowel symptoms, n (%)	2 (7.4)	10 (3.3)	0.260
Infertility, n (%)	1 (4.8)	6 (3.8)	0.590

TVS: transvaginal sonography; SD: standard deviation.

Interestingly, one patient in this series was asymptomatic and had no sonographic signs suggestive of endometriosis in her first rTVS. Twenty-two months later, she was referred to another TVS with severe dysmenorrhea, and the second scan showed a retrocervical nodule.

## DISCUSSION

This seems to be the first study to evaluate the prevalence of sonographic signs suggestive of endometriosis in women who underwent rTVS for gynecological evaluation with no clinical suspicion of endometriosis. We found lesions suggestive of DIE and endometriomas in 8% of participants, which is higher than the estimated prevalence of DIE in women of reproductive age (1-2%).^(21)^ The retrocervical region was the most frequently involved site (85%), followed by ovarian endometriomas. Since endometriomas are easier to detect using rTVS, we believe that most women with endometriomas might have already been detected and proceeded with further investigation or surgery.

Although visual inspection or histological identification is the only currently available diagnostic test for endometriosis, imaging methods have been adopted as valuable tools for complementary clinical assessment and to assist in treatment planning.^(8,11)^ As TVS is an accessible, inexpensive, and well-tolerated imaging modality, this procedure is considered the first imaging choice for gynecological evaluation. The sensitivity of TVS for the detection of endometriosis varies between 50% and 100% in the published literature, and depends on the anatomical site involved.^(9,22)^ In addition, its discovery also relies on the experience of the examiner. Conversely, the specificity is very high for experienced hands, reaching 94-100%.^(9,13,14,21,23,24)^ Thus, the disease cannot be excluded with confidence if there are no sonographic signs. However, the presence of a suggestive sonographic lesion is highly predictive of endometriosis.

In 2016, the International Deep Endometriosis Analysis (IDEA) group published a consensus report on the sonographic approach for evaluating the pelvis of women with suspected endometriosis.^(16)^ Even though our scans were performed before the publication of this consensus, we followed some steps that later were considered important in the evaluation of the pelvis proposed by the IDEA group, *i.e.,* evaluation of the uterus and adnexa and the active search for signs suggestive of DIE nodules in the anterior and posterior compartments. Because the patients in our study were not referred for further investigation of clinically suspected endometriosis but rather for a routine annual pelvic evaluation, the evaluation of “soft markers” and the “sliding sign”, which are steps of the IDEA evaluation, was not systematically performed to avoid patient discomfort and unnecessary prolongation of the scan time in asymptomatic or oligosymptomatic women. We acknowledge that the prevalence of sonographic signs of DIE in the group studied might have been underestimated.

Almost 50% of the women with signs suggestive of endometriosis in our sample were asymptomatic. This is an important finding since it has been previously estimated that only ~5% of women with DIE are asymptomatic.^(21)^ We also found suggestive signs of DIE, including bowel disease in oligosymptomatic patients. In our study, the presence of symptoms did not correctly differentiate women with and without sonographic signs suggestive of endometriosis. Notably, the infertility rate in our sample (2.1%) was much lower than expected,^(25)^ even for women without endometriosis (~15%). This results are probably justified because only approximately 50% of our cohort had already attempted to conceive.

There are limitations to our study. Since the study included just imaging data, we had no access to gynecological follow-up of patients with signs of endometriosis at rTVS. Therefore, we could not estimate the probability of endometriosis progression or the occurrence of symptoms. In addition, because all examinations were performed without bowel preparation and only the rectum and sigmoid were examined, small lesions and those in other bowel locations may have remained undetected. Finally, since the sensitivity of TVS to detect endometriosis is lower than its specificity, in our study the actual disease prevalence may have been underestimated.

However, it is important to highlight that one of the strengths of the present study is that all examinations were performed by the same radiologist trained to detect and stage DIE, and routinely search for signs of the disease. Fraser et al.^(10)^ highlighted the importance of ultrasound in the detection of endometriosis when performed by an experienced radiologist. The authors retrospectively compared the findings of rTVS and expert-guided TVS (both performed before surgery) in 40 women with chronic pelvic pain and later histological confirmation of endometriosis. Routine TVS detected signs of endometriosis in only 10 patients (all with endometriomas; extraovarian endometriosis lesions were not detected). On the other hand, expert-guided TVS accurately detected endometriosis in 31 women. This might be one of the reasons why the diagnosis is often delayed.^(26–28)^

Although our findings were not surgically confirmed systematically, we can assume that the majority of the lesions observed in TVS would correspond to actual endometriosis lesions given the high specificity of TVS. This study sheds light on the validity of using TVS performed by a trained radiologist in prospective studies as a robust tool for investigating the natural history of the disease in asymptomatic or oligosymptomatic women.

Additionally, even though those women might never develop moderate or severe symptoms or impairment in their quality of life, to estimate the frequency of sonographic signs suggestive of endometriosis among asymptomatic women is important as it represents the “noise” that should be considered in studies associating sonographic findings with endometriosis symptoms or disease progression. We believe that this information may contribute to the understandings of this disease, especially among asymptomatic and oligosymptomatic patients.

## CONCLUSION

Routine transvaginal sonography is increasingly proving to be a valuable tool for detecting deep infiltrative endometriosis even in clinically unsuspected diseases. Here, we showed that transvaginal sonography performed by an experienced radiologist can detect signs of extraovarian endometriosis (more specifically, deep infiltrative endometriosis). Therefore, imagers should be more familiar with these findings. A prospective study is necessary to address this question. There is no information regarding the relevance of these findings in asymptomatic women. We believe that this would be an important step not only to further investigate the natural history and progression of the disease, but also to develop a method to detect endometriosis in women before the onset of symptoms. This method would benefit patients who may receive counseling and early treatment.
